# Convolution of Barker and Mutually Orthogonal Golay Complementary Codes for Ultrasonic Testing

**DOI:** 10.3390/s25165007

**Published:** 2025-08-13

**Authors:** Chengxiang Peng, Paul Annus, Marek Rist, Raul Land, Madis Ratassepp

**Affiliations:** 1 Department of Civil Engineering and Architecture, Tallinn University of Technology, 19086 Tallinn, Estonia; chengxiang.peng@taltech.ee; 2 Thomas Johann Seebeck Department of Electronics, Tallinn University of Technology, 19086 Tallinn, Estonia

**Keywords:** nondestructive testing, ultrasonic testing, coded signals, time efficiency

## Abstract

Ultrasonic testing (UT) is a vital nondestructive testing (NDT) technique used to evaluate the integrity of materials and structures. However, conventional excitation signals often suffer from significant attenuation in highly attenuative materials, resulting in low signal energy and poor signal interpretation. Coded excitation techniques, such as the Barker code and the complementary Golay code (CGC), have been used to enhance signal energy and signal-to-noise ratio. Yet, Barker codes are limited by short sequence lengths, while CGC requires two transmission events, reducing time efficiency. This paper proposes a novel excitation method: the Barker-convolved mutually orthogonal Golay complementary code (BMOGCC). By convolving the Barker code with the mutually orthogonal Golay complementary code (MOGCC), BMOGCC combines the advantages of both, including flexibility in code length, improved signal amplitude, low sidelobe levels, and enhanced time efficiency. Performance was evaluated using numerical simulations and laboratory experiments, with key indices including the peak sidelobe level (PSL), mainlobe gain (MG), and temporal resolution. The results show that BMOGCC achieves a significantly higher MG than either the Barker code or MOGCC alone while maintaining a low PSL and preserving the temporal resolution. These findings demonstrate that BMOGCC is effective and efficient for coding excitation signals in ultrasonic testing, offering improved signal quality and measurement time efficiency.

## 1. Introduction

Nondestructive testing (NDT) enables the inspection of materials, components, and structures without causing damage or interrupting their operation and is essential to ensure the safety and integrity of civil structures such as metal plates and pipelines [1,2,3]. Ultrasonic testing (UT), a critical NDT technique, is widely used in a number of fields, such as civil engineering, aerospace, and the energy industry, due to its high efficiency and ability to detect various types of defects [4,5,6]. UT involves the generation, transmission, and reception of ultrasonic waves that interact with defects in the material. The health condition of the material is then assessed by processing the received signals. Consequently, the quality of the received signals is vital to the reliability of UT.

Ultrasonic signals are vulnerable to attenuation and noise contamination in practical measurement environments. For example, signals can be heavily distorted due to long-distance propagation, such as in rail inspections [7]. In UT, signal energy is a valuable resource. High energy enables ultrasonic waves to propagate over longer distances, allowing the detection of material defects located farther from the ultrasonic transducer. One approach to increasing the signal energy under adverse measurement conditions is to raise the signal voltage. However, high voltage is often prohibited for various reasons, such as in flammable environments and due to limitations of data acquisition systems [8]. Another approach to increasing the signal energy is to extend the signal duration. However, simply extending the duration degrades the temporal resolution and creates strong sidelobes [9].

**Figure 1:**
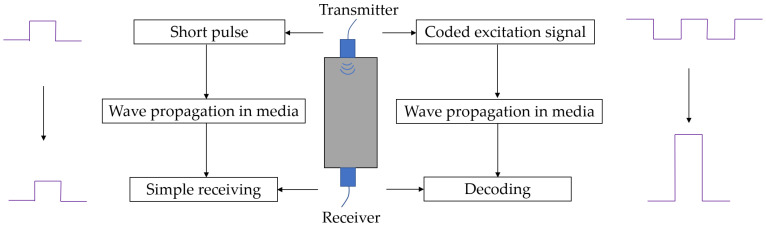
Schematic diagram of a UT system using a coded excitation signal.

Coded signals employ extended coded pulses to increase signal energy without raising pulse voltage, thereby enhancing measurement reliability and safety. For a signal received from a UT system using a coded excitation signal, a decoding process commonly known as pulse compression is needed to compress the long coded signal into a narrow peak, thus enhancing signal amplitude without sacrificing temporal resolution [10,11]. These advantages make coded excitation signals widely used in UT, particularly for testing highly attenuated materials and performing long-distance inspections. Figure 1 presents a UT system using a coded excitation signal.

Coded signals were initially used in radar systems and later introduced into ultrasonic systems. In 1979, Takeuchi was the first to propose their application in medical ultrasound systems, emphasizing the benefits of spreading signal energy over time and highlighting the time-bandwidth product for improving the signal-to-noise ratio (SNR) [12]. Based on the modulation type of the carrier wave used to construct coded signals, coding methods can be categorized into frequency modulation (FM), amplitude modulation (AM), and phase modulation (PM). FM signals have a varying instantaneous frequency, resulting in better pulse compression. Numerous FM techniques, including linear FM, have been applied in UT. Linear FM is the simplest case, in which the instantaneous frequency changes linearly over time. Gan et al. demonstrated an SNR improvement in measurements of metal plates using linear FM [13]. AM encodes the signal by modulating the amplitude of the carrier wave. Eckersley et al. investigated the effectiveness of AM for imaging at low acoustic power [14]. In PM, the carrier wave retains constant amplitude and frequency, while modulation occurs through controlled phase shifts. These phase shifts facilitate more effective pulse compression by suppressing sidelobes. Various PM codes have been employed in UT, with Barker codes and the complementary Golay code (CGC) among the most commonly used. Barker codes are valued for their simplicity and relatively low sidelobe levels. For example, Jiang et al. used Barker codes to improve the SNR in the inspection of highly attenuated wood materials [15]. Using Barker codes, Xia et al. achieved greater effectiveness than average filtering in measuring material thickness [16]. CGC is capable of eliminating sidelobes, which are inevitable in Barker codes. Garcia-Rodriguez et al. employed CGC in air-coupled ultrasonic Lamb-wave systems to achieve more precise time-of-flight calculations [17]. Germano et al. applied CGC in the ultrasonic total focusing method, obtaining greater penetration depth and improved image quality [18].

However, Barker codes are limited to only seven unique sequences, with a maximum length of 13. This restricts their effectiveness in enhancing signal amplitude and SNR, and limits the flexibility to choose a suitable code length for practical measurements [19,20]. Although CGC shows excellent performance in sidelobe suppression and signal amplitude enhancement, it requires two transmission events, thereby halving the time efficiency of measurements [21,22]. Recent studies have attempted to address the limitations of Barker codes and CGC. Kim et al. proposed the Barker-convolved Golay code (BCGC), formed by convolving Barker codes with CGC, which expands code length flexibility and increases signal amplitude [23]. However, the study used only the 3-bit Barker code to construct the BCGC. Zeng et al. conducted a more comprehensive theoretical study covering all Barker code lengths [19]. Later, Zeng et al. explored the performance of BCGC for pipeline inspection [24]. BCGC provides more options in code length and improves signal amplitude but still suffers from the same low time efficiency as CGC. Yang et al. developed multivariate codes (MC), constructed from multiple convolutions of Barker codes and CGC, reducing the number of transmissions to one [25]. However, to achieve the same peak sidelobe level, MC requires nearly double the code length of BCGC, again compromising time efficiency. Although still requiring two transmission events, the mutually orthogonal Golay complementary code (MOGCC) enables simultaneous transmission from two transducers, thus achieving twice the data acquisition efficiency compared to CGC [26,27]. Although MOGCC addresses the time efficiency problem, its improvement in signal amplitude remains equivalent to that of CGC and is still inferior to BCGC. Therefore, the limitations of Barker codes and CGC have only been partially addressed, and more research is needed to achieve performance improvement.

In this paper, the Barker-convolved mutually orthogonal Golay complementary code (BMOGCC) is proposed for UT. BMOGCC combines the advantages of Barker codes and MOGCC, offering improved performance compared to conventional Barker codes, CGC, and recent developments like MOGCC, BCGC, and MC. Specifically, BMOGCC provides a higher signal amplitude than Barker codes, CGC, and MOGCC. In addition, BMOGCC achieves better time efficiency than the recently developed BCGC and MC. Moreover, BMOGCC offers more flexible code lengths to suit different testing requirements. To evaluate the effectiveness of BMOGCC, ultrasonic wave propagation in a steel block was simulated using two-dimensional (2D) finite element (FE) modeling, and corresponding laboratory experiments were conducted. The performance of BMOGCC was analyzed and compared with that of Barker codes and MOGCC. The rest of this paper is organized as follows. Section 2 introduces Barker codes and MOGCC, presents the derivation of BMOGCC, and defines the performance evaluation criteria. Section 3 validates the performance of BMOGCC through numerical simulations. Section 4 demonstrates the performance of BMOGCC on the basis of experimental data. Section 5 discusses the results, and Section 6 concludes this paper.

## 2. Methodology

### 2.1. Barker Codes

BMOGCC is formed by convolving the Barker code with MOGCC, thereby inheriting certain properties of Barker codes. Barker codes are widely used binary phase sequences due to their low sidelobe level after decoding. For a Barker-coded sequence *B* of length $N_{B}$ composed of B(1),B(2),B(3),…,B(N), its autocorrelation, namely the decoded sequence $C_{B}$, is as follows [28]:

(1) $$C_{B}\left(j\right)=\sum_{i=1}^{N_{B}}B\left(i\right)B\left(i+j\right)=\left\{\begin{array}{cc} N_{B}, & j=0\\ 0\;or\;\pm 1, & 0<j<N_{B}.\\ 0, & j\geq N_{B} \end{array}\right.$$

Equation (1) shows that the autocorrelation peak value is equal to the coded sequence length $N_{B}$, and the sidelobe level is limited to 1, demonstrating the effectiveness of Barker codes in pulse compression. It is also evident that a longer sequence results in better compression performance. However, the maximum length of Barker codes is 13, which limits the maximum value of $C_{B}$ to 13. Table 1 lists Barker codes of different lengths.

### 2.2. MOGCC

**Figure 2:**
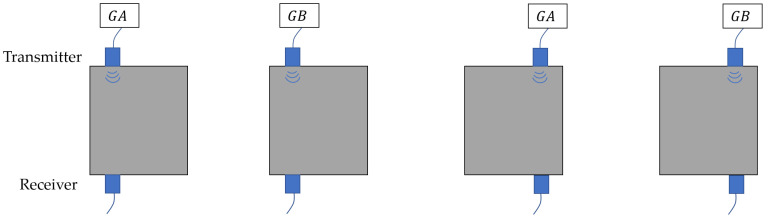
Use of the complementary Golay code (CGC) for ultrasonic testing.

When using CGC, a transducer requires two transmissions to achieve zero sidelobes, resulting in low time efficiency. This issue becomes more serious when inspecting large areas that require transducer relocation or the use of multiple transducers. For example, the CGC consisting of two coded sequences GA and GB requires four transmission events to complete inspection at two locations, as illustrated in Figure 2. MOGCC, which consists of two pairs of mutually orthogonal complementary sequences, doubles time efficiency by enabling simultaneous transmission from two transducers, with each still transmitting twice [26], resulting in two transmission events in total. Due to the orthogonality between the pairs, the received signal can be separated into components corresponding to each individual transmitter. For the MOGCC composed of two pairs MX and MY with length $N_{M}$, where MX consists of two sequences $MX_{1}$ and $MX_{2}$, and MY consists of $MY_{1}$ and $MY_{2}$, the combined autocorrelations are given as

(2) $$C_{X}\left(j\right)=C_{X1}\left(j\right)+C_{X2}\left(j\right)=MX_{1}*MX_{1}\left(-i\right)+MX_{2}*MX_{2}\left(-i\right)=2N_{M}\delta \left(j\right),$$

(3) $$C_{Y}\left(j\right)=C_{Y1}\left(j\right)+C_{Y2}\left(j\right)=MY_{1}*MY_{1}\left(-i\right)+MY_{2}*MY_{2}\left(-i\right)=2N_{M}\delta \left(j\right),$$

where $C_{X}$ and $C_{Y}$ denote the combined autocorrelations of MX and MY, respectively. $C_{X1}$, $C_{X2}$, $C_{Y1}$, and $C_{Y2}$ are autocorrelations for $MX_{1}$, $MX_{2}$, $MY_{1}$, and $MY_{2}$, respectively. The symbol ∗ denotes the convolution operation, and $MY_{1}\left(-i\right)$ is the time inverse of $MY_{1}$. Furthermore, the sum of the cross-correlations between the two pairs is given by

(4) $$MX_{1}*MY_{1}\left(-i\right)+MX_{2}*MY_{2}\left(-i\right)=MY_{1}*MX_{1}\left(-i\right)+MY_{2}*MX_{2}\left(-i\right)=0.$$

**Figure 3:**
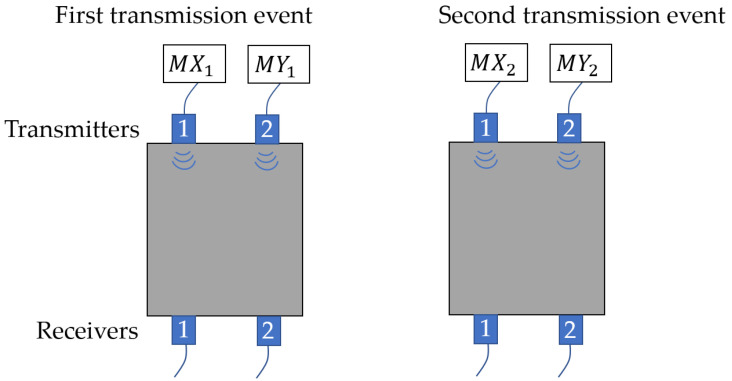
Implementation of MOGCC in a UT system.

Using Equations (2)–(4), MOGCC can be applied in a UT system that includes two transmitters and two receivers, where $MX_{1}$ and $MX_{2}$ are two transmitted sequences for one transmitter, and $MY_{1}$ and $MY_{2}$ for another. Figure 3 illustrates a UT system using MOGCC, where $MX_{1}$ and $MY_{1}$ are sent simultaneously in the first transmission event, and $MX_{2}$ and $MY_{2}$ are sent simultaneously in the second transmission event.

Although the receivers capture overlapping signals from both transmitters, the signals can be separated so that receiver 1 extracts only the signal from transmitter 1, and receiver 2 from transmitter 2. Taking receiver 1 as an example, to extract the signal originating from transmitter 1, we have

(5) $$\begin{array}{cc} C_{XY} & =\left(MX_{1}+MY_{1}\right)*MX_{1}\left(-i\right)+\left(MX_{2}+MY_{2}\right)*MX_{2}\left(-i\right)\\ & =MX_{1}*MX_{1}\left(-i\right)+MX_{2}*MX_{2}\left(-i\right)+MY_{1}*MX_{1}\left(-i\right)+MY_{2}*MX_{2}\left(-i\right)\\ & =MX_{1}*MX_{1}\left(-i\right)+MX_{2}*MX_{2}\left(-i\right)\\ & =2N_{M}\delta \left(j\right), \end{array}$$

where $C_{XY}$ is the separation result, and also the decoded result, in receiver 1. The decoded sequence $C_{XY}$ has a peak amplitude of $2N_{M}\delta \left(j\right)$ and zero sidelobes, illustrating the effectiveness of MOGCC in pulse compression. In addition, the separation result for receiver 2 is also $2N_{M}\delta \left(j\right)$. Moreover, CGC and MOGCC yield the same peak amplitude $C_{XY}$ after decoding, provided that they have the same code length. Comparing Figure 2 and Figure 3, it is evident that MOGCC requires only two transmission events to complete inspections at two locations, which is half the time required by CGC. Table 2 lists the 4-bit MOGCC (M4) and the 8-bit MOGCC (M8), where M4 consists of $M4X_{1}$, $M4X_{2}$, $M4Y_{1}$, and $M4Y_{2}$, and M8 consists of $M8X_{1}$, $M8X_{2}$, $M8Y_{1}$, and $M8Y_{2}$.

### 2.3. BMOGCC

**Figure 4:**
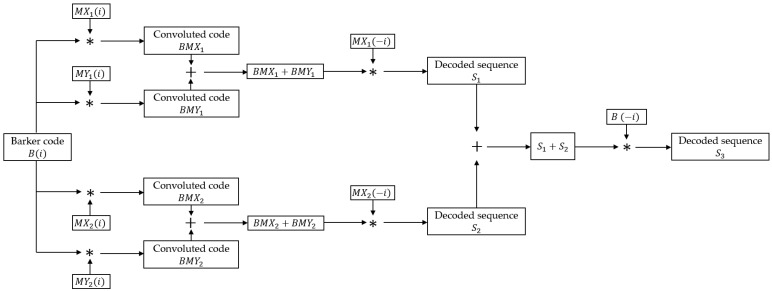
Flow of generating and decoding BMOGCC.

BMOGCC is formed by convolving the Barker code and MOGCC, thereby retaining the advantageous properties of both. The process of generating and decoding BMOGCC is illustrated in Figure 4.

The process consists of five steps. The first step involves the generation of BMOGCC, while the remaining four steps are dedicated to decoding.

Step 1: The four MOGCC sequences $MX_{1},\;MX_{2},\;MY_{1}$, and $MY_{2}$ are each convolved with the Barker code. The resulting sequences, denoted as $BMX_{1},\;BMX_{2},\;BMY_{1}$, and $BMY_{2}$, make up the BMOGCC. These codes are calculated as follows:

(6) $$BMX_{1}=MX_{1}*B,\;BMX_{2}=MX_{2}*B,\;BMY_{1}=MY_{1}*B,\;BMY_{2}=MY_{2}*B.$$

Step 2: A summation is performed between $BMX_{1}$ and $BMY_{1}$, and separately between $BMX_{2}$ and $BMY_{2}$.

Step 3: Each summation result from Step 2 is convolved with the time-reversed versions of MX1 and MX2, respectively, yielding the decoded sequences $S_{1}$ and $S_{2}$. The convolution operations are written as

(7) $$\begin{array}{cc} S_{1} & =\left(BMX_{1}+BMY_{1}\right)*MX_{1}\left(-i\right)=B\left(i\right)*\left(MX_{1}+MY_{1}\right)*MX_{1}\left(-i\right),\\ S_{2} & =\left(BMX_{2}+BMY_{2}\right)*MX_{2}\left(-i\right)=B\left(i\right)*\left(MX_{2}+MY_{2}\right)*MX_{2}\left(-i\right). \end{array}$$

Step 4: The decoded sequences $S_{1}$ and $S_{2}$ are summed.

Step 5: The sum of $S_{1}$ and $S_{2}$ is convolved with the time-reversed Barker code B(−i), resulting in the final decoded sequence $S_{3}$, which is written as

(8) $$\begin{array}{cc} S_{3} & =\left(S_{1}+S_{2}\right)*B\left(-i\right)\\ & =B\left(i\right)*\left[\left(MX_{1}+MY_{1}\right)*MX_{1}\left(-i\right)+\left(MX_{2}+MY_{2}\right)*MX_{2}\left(-i\right)\right]*B\left(-i\right)\\ & =B\left(i\right)*B\left(-i\right)*(MX_{1}*MX_{1}\left(-i\right)+MX_{2}\left(-i\right)*MX_{2})\\ & =C_{B}*C_{XY}. \end{array}$$

From the steps above, it is clear that the length of BMOGCC is $N_{BM}=N_{B}+N_{M}-1$. Equation (8) demonstrates that the decoded sequence $S_{3}$ is the convolution of the decoded Barker code and MOGCC. Therefore, BMOGCC achieves a higher compression peak than either the Barker code or MOGCC alone and reaches the same compression peak as BCGC, which is formed by convolving the Barker code with CGC [23]. Moreover, BMOGCC offers the same time efficiency as MOGCC, which is twice that of BCGC and CGC.

**Figure 5:**
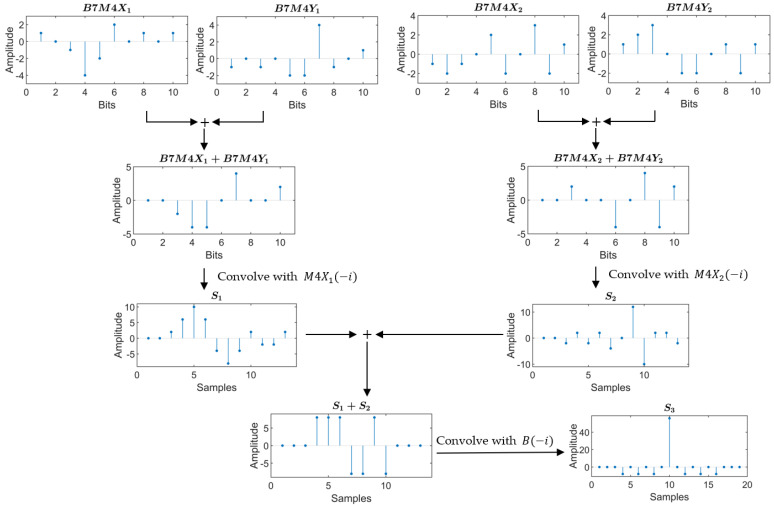
Decoding process for B7M4.

Figure 5 presents the decoding results of B7M4, which is the convolution of the 7-bit Barker code and the M4 from Table 2. The four sequences that make up B7M4 are denoted as $B7M4X_{1}$, $B7M4X_{2}$, $B7M4Y_{1}$, and $B7M4Y_{2}$. The decoded sequence $S_{3}$ of the 10-bit B7M4 clearly shows a main peak amplitude of 56, which is significantly higher than that of the 13-bit Barker code or the 16-bit MOGCC. This highlights the superior signal-amplitude enhancement capability of BMOGCC compared to both the Barker code and MOGCC.

### 2.4. Performance Evaluation of Codes

In practical measurements, the carrier wave is modulated by the code to generate the coded excitation signal, where each bit of the code modulates the carrier wave. The modulated excitation signal $S_{c}$ is defined as

(9) $$S_{c}=\sum_{i=1}^{N}c\left(i\right)w\left(t-i\cdot T\right),\;0\leq t\leq T$$

where c(i) is the i-th bit of the code and w(t) is the carrier wave.

The performance of coded excitation signals, or equivalently, of codes themselves, is evaluated using several indices, including the peak sidelobe level (PSL), integrated sidelobe level, mainlobe gain (MG), and mainlobe level. Definitions may vary across studies [10,19,24,25,26,29]. Among these indices, the PSL and MG are two of the most critical for evaluating code performance. The PSL is defined as the logarithmic ratio of the mainlobe peak to the maximum sidelobe peak of the decoded signal [29]:

(10) $$PSL=20log10(\frac{A_{m}}{A_{s}}),$$

where $A_{m}$ is the amplitude of the mainlobe peak and $A_{s}$ is the maximum sidelobe amplitude. The PSL quantifies the code’s ability to suppress self-noise introduced in the decoding process. The MG is the logarithmic ratio of the mainlobe peak amplitude of the decoded signal obtained using a coded excitation signal to that obtained using a 1-bit pulse (unmodulated carrier wave) [24]:

(11) $$MG=20\log10(\frac{A_{m}}{A_{p}}),$$

where $A_{p}$ is the peak amplitude of the compressed 1-bit pulse. The MG represents the ability of a code to enhance signal amplitude. Table 3 lists the theoretical PSL and MG values. Note that the PSL is not defined for CGC and MOGCC because they ideally produce zero sidelobes.

**Figure 6:**
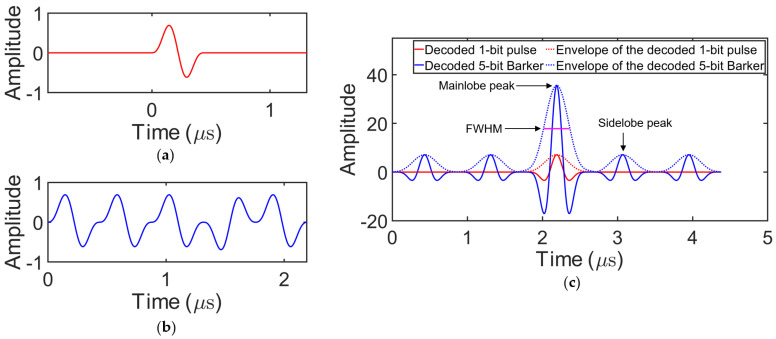
(**a**) 1-bit pulse. (**b**) 5-bit Barker-coded excitation signal. (**c**) Decoded results.

In addition to the PSL and MG, the temporal resolution, estimated by the full width at half maximum (FWHM) of the decoded signal envelope, is important in certain NDT imaging methods, such as the total focusing method [30,31]. Figure 6c shows the decoded signals for a 1-bit pulse and the 5-bit Barker-coded signal, in which the mainlobe, sidelobes, and temporal resolution are clearly characterized. The 1-bit pulse is a 2.25 MHz sine wave windowed by a Hanning window and is shown in Figure 6a. The 5-bit Barker-coded excitation signal generated using this 1-bit pulse as the carrier wave is shown in Figure 6b.

## 3. Numerical Simulations

### 3.1. Finite Element Modeling

**Figure 7:**
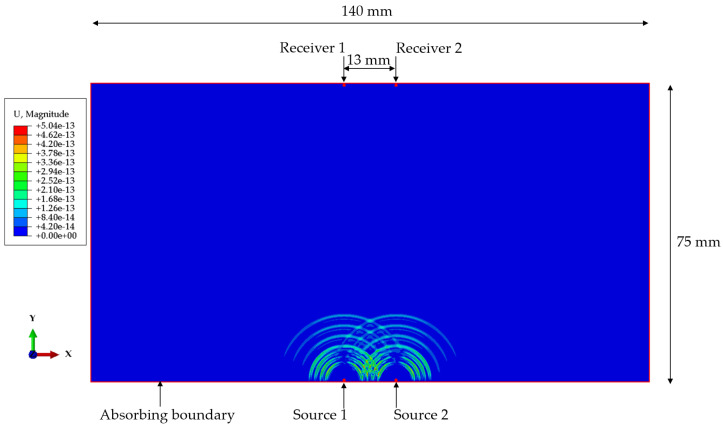
Geometry of the simulation model and the displacement of the propagating waves from two sources, shown at 3 μs.

Simulations were conducted using the software ABAQUS version 2021 [32] to evaluate the performance of the coded signals. A 2D finite element model (Figure 7) was developed, consisting of a steel plate of size 75 mm × 140 mm, with two parallel excitation source nodes and two parallel receiver nodes aligned accordingly. The vertical distance between the source nodes and receiver nodes was 74 mm. The material properties of the steel used in the simulations are listed in Table 4. The model was meshed using the CPE4R element type. To prevent reflections at the model boundaries, a layer of CINPE4 elements was applied outside the model perimeter. The excitation was applied as a vertical force at the source nodes, and the vertical displacements were recorded at the receiver nodes.

The 1-bit pulse used to construct the coded excitation signals is shown in Figure 6a, with a center frequency of 2.25 MHz, matching that of the ultrasonic transducers used in the experiments. In ABAQUS, the maximum frequency that can be simulated is limited by the element size; higher frequencies require smaller elements [33]. Coded excitation signals may contain high-frequency components that are several times the center frequency because of the phase modulation. To ensure that the simulations were convergent, an element size of 0.025 mm was used, which corresponds to approximately 102 elements per wavelength. If a larger element size, such as 0.05 mm, is used, the resulting wave may fail to converge, leading to incorrect signals for further analysis.

A series of simulations was performed using different excitation signals to evaluate the performance of BMOGCC. Specifically, codes B5M4, B5M8, B7M4, and B7M8 were evaluated. For comparison, three Barker codes (from Table 1), as well as M4 and M8 (from Table 2), were tested. In addition, the 1-bit pulse was included as a reference excitation signal for calculating the mainlobe gain. Since both BMOGCC and MOGCC require two transmission events, two separate simulations were conducted for each of these codes. For simulations involving two sources, the excitations were applied simultaneously. Table 5 lists the excitation signals applied to each source across all simulations. Figure 7 also illustrates the displacement field for simulation 5 as an example, where the faster-propagating compression wave was used in the decoding, while the slower-propagating shear wave was filtered out by a time window.

### 3.2. Results of Decoding

**Figure 8:**
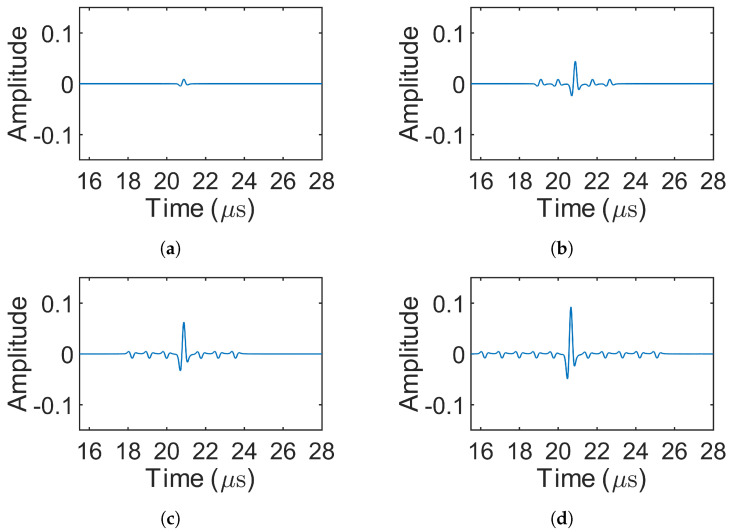
Decoded signals for the 1-bit pulse and the Barker codes (simulations 1–4). (**a**–**d**) 1-bit pulse, 5-bit Barker, 7-bit Barker, and 11-bit Barker, respectively.

**Figure 9:**
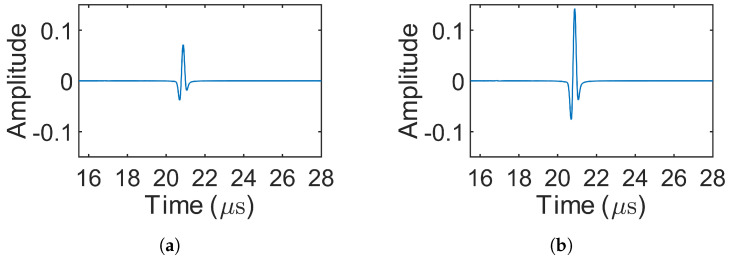
Decoded signals for MOGCC (simulations 5–8). (**a**,**b**) M4 (simulations 5–6) and M8 (simulations 7–8), respectively.

**Figure 10:**
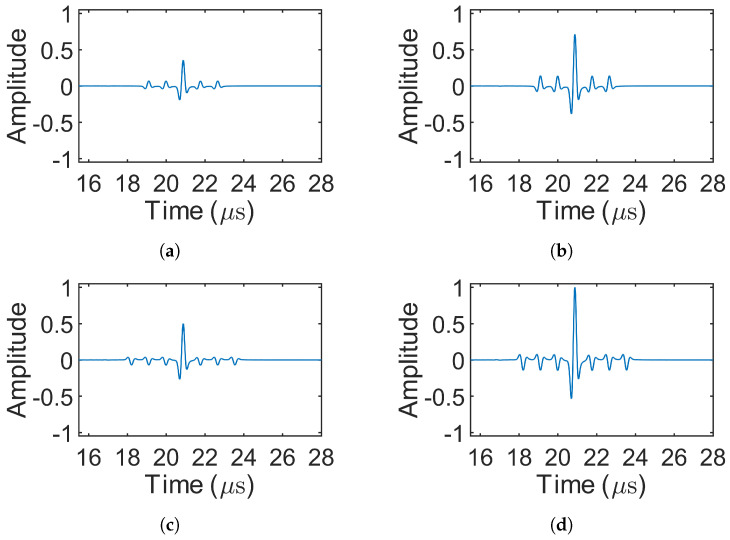
Decoded signals for BMOGCC (simulations 9–16). (**a**–**d**) B5M4 (simulations 9–10), B5M8 (simulations 11–12), B7M4 (simulations 13–14), and B7M8 (simulations 15–16), respectively.

Figure 8 presents the decoded signals for the 1-bit pulse (simulation 1) and the three Barker codes (simulations 2–4), where only source 1 was excited. In simulations involving MOGCC or BMOGCC, only the decoded signals from receiver 1 are shown, as the decoded signals from both receivers were symmetric. Figure 9 shows the decoded signals for MOGCC (simulations 5–8), with each decoded signal based on the signals received from two transmission events. Figure 10 displays the decoded signals for BMOGCC (simulations 9–16), including B5M4, B5M8, B7M4, and B7M8. The amplitude of the decoded signals was normalized to the maximum amplitude value of all decoded signals to clearly illustrate the amplitude difference among the codes.

It can be observed in Figure 8, Figure 9 and Figure 10 that the signal amplitudes varied depending on the code type, reflecting the distinct properties of each coding scheme. As shown in Figure 8, the amplitude of the decoded signals increased progressively with the length of the Barker code, and visually uniform sidelobes were present due to the inherent correlation characteristics of these codes. Figure 9 shows that MOGCC produced no sidelobes and that M8 exhibited a higher amplitude than M4, attributed to its longer code length. In contrast, Figure 10 demonstrates that BMOGCC achieved a markedly higher decoded amplitude compared to both MOGCC and the Barker codes. This result aligns with Equation (8), which expresses the BMOGCC-decoded signal as the convolution of the MOGCC- and Barker-code-decoded signals.

## 4. Experimental Measurements

### 4.1. Experimental Setup

**Figure 11:**
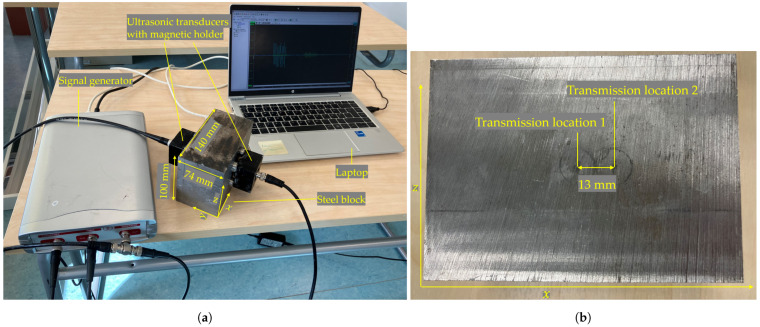
Experimental setup. (**a**) Measurement setup including ultrasonic transducers, a signal generator, and a laptop. (**b**) Transmission locations on the steel block.

To further evaluate the performance of the coded signals, experimental measurements were carried out to ensure that factors such as the transducer response and data acquisition (DAQ) system characteristics did not significantly impact the results. The experimental setup, shown in Figure 11a, included a 140 mm × 74 mm × 100 mm steel block, two ultrasonic transducers, and a DAQ system. The transducers (M1036, Olympus, Hamburg, Germany) had a center frequency of 2.25 MHz and were attached to the steel block using magnetic holders. Ultrasonic coupling gel was applied to ensure good acoustic contact. The DAQ system included a signal generator (HS805, TiePie, Sneek, The Netherlands) with an 8-bit amplitude resolution. It was connected to a computer via USB and operated using the TiePie oscilloscope software version 1.33.2.0/0.5.6.2, which allowed configuration of the excitation signals, voltage, and sampling rate.

A through-transmission scheme was used to capture the ultrasonic compression waves, with the transducers placed on opposite sides of the steel block. The transmission and reception locations mirrored the simulation setup shown in Figure 7. As in the simulations, the setup included two transmission and two receiving locations, requiring four transducers in total. The distance between the two transmission locations was 13 mm, which is equal to the diameter of the ultrasonic transducers (Figure 11b). However, due to the availability of only two suitable transducers, a practical workaround was adopted: one transducer was moved from one transmission location to the other, and the signals received at the same receiving location were added. This approach effectively replicated the signal that would have been obtained if both transmitters had operated simultaneously.

The excitation signals used in the experiments were identical to those used in the simulations, resulting in a total of 16 experimental trials. The settings of the excitation signals for experiments 1–16 were identical to those of simulations 1–16.

### 4.2. Results of Decoding

**Figure 12:**
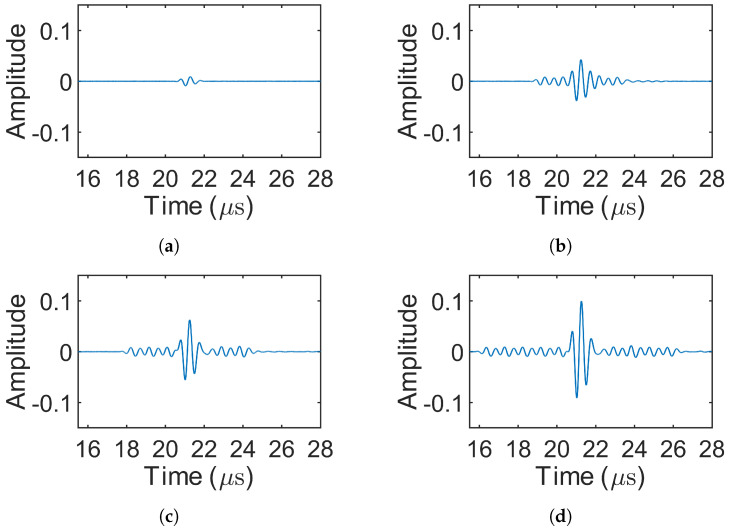
Decoded signals for experiments 1–4. (**a**–**d**) 1-bit pulse, 5-bit Barker, 7-bit Barker, and 11-bit Barker, respectively.

**Figure 13:**
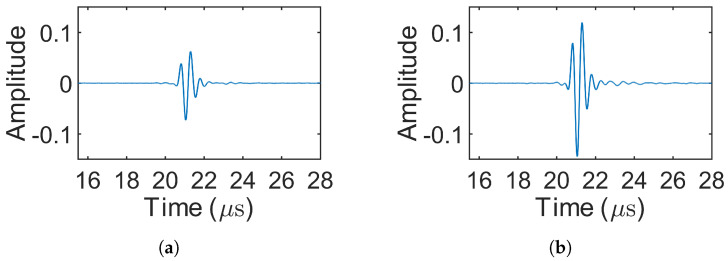
Decoded signals for MOGCC (experiments 5–8). (**a**,**b**) Decoded signal of M4 (experiments 5–6) and M8 (experiments 7–8), respectively.

**Figure 14:**
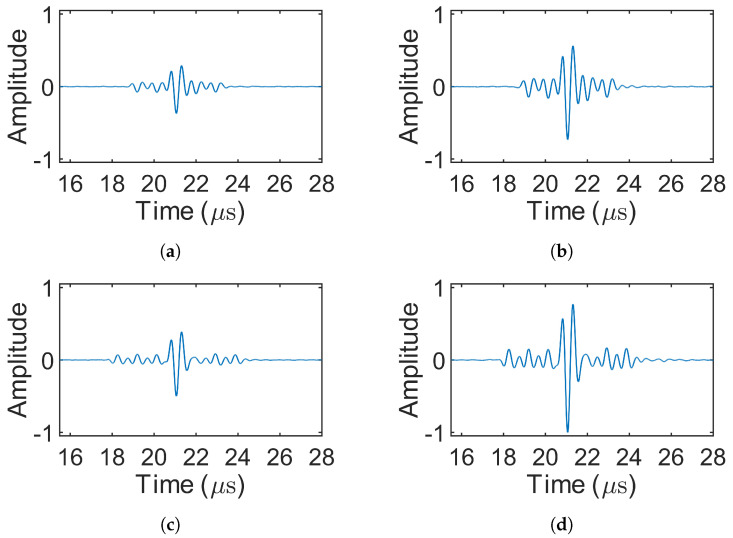
Decoded signals for BMOGCC (experiments 9–16). (**a**–**d**) B5M4 (experiments 9–10), B5M8 (experiments 11–12), B7M4 (experiments 13–14), and B7M8 (experiments 15–16), respectively.

As in the simulations, only the decoded signals from receiving location 1 are presented, since the decoded signals from both locations were symmetric. Figure 12 shows the decoded signals for the 1-bit pulse (experiment 1) and the Barker codes (experiments 2–4). Figure 13 displays the decoded signals for MOGCC (experiments 5–8). Figure 14 presents the decoded signals for BMOGCC (experiments 9–16), including B5M4, B5M8, B7M4, and B7M8. The decoded signals were normalized using the same method as in the simulations, dividing by the global maximum amplitude, to facilitate comparison across different codes.

The amplitudes of the decoded signals were found to be consistent with the simulation results. Figure 12 shows that the amplitude increased with the length of the Barker codes, accompanied by low-level sidelobes. As depicted in Figure 13, M8 yielded a higher amplitude than M4, owing to its doubled code length. Figure 14 demonstrates that BMOGCC produced a higher decoded signal amplitude than both MOGCC and the Barker codes. However, the sidelobes were less uniform than those seen in the simulations, likely due to factors such as modulation by the transducer’s impulse response and increased noise resulting from the limited amplitude resolution of the DAQ system.

## 5. Discussion

By comparing Figure 8, Figure 9 and Figure 10 and Figure 12, Figure 13 and Figure 14, it is evident that all coded signals yielded higher decoded mainlobe amplitudes than the 1-bit pulse, with BMOGCC yielding the highest amplitudes among all the codes evaluated. The experimental results (Figure 12, Figure 13 and Figure 14) showed that the mainlobe amplitudes of the decoded signals were approximately consistent with those of the corresponding simulation results. These observations confirm the capability of BMOGCC to significantly enhance the mainlobe amplitude.

**Figure 15:**
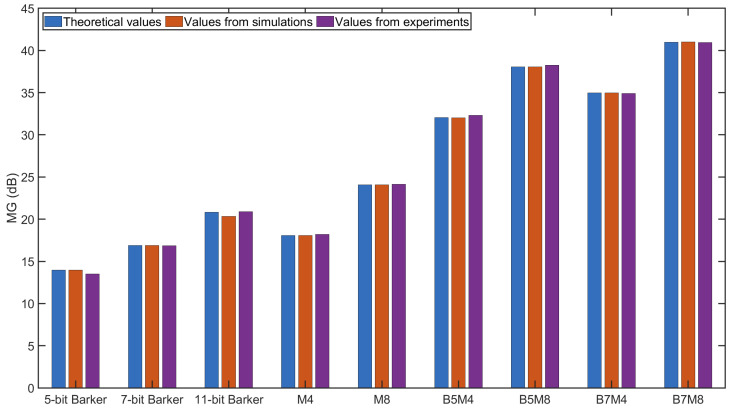
MG values of different codes.

Two key indicators, the peak sidelobe level (PSL) and the mainlobe gain (MG), were calculated and compared to evaluate the code performance, based on the decoded signals from Figure 8, Figure 9 and Figure 10 and Figure 12, Figure 13 and Figure 14. The MG values from both the simulations and experiments are shown in Figure 15, along with the corresponding theoretical values based on Table 2. The measured MG values closely matched the theoretical predictions. Notably, BMOGCC achieved significantly higher MG values than either the Barker codes or MOGCC, reaching maximum values for B7M8. Moreover, the MG value of each BMOGCC was approximately equal to the sum of the MG values of the corresponding Barker and MOGCC in its construction, as expected. For instance, in the experiments, the MG values of B7M4, B7, and M4 were 34.90 dB, 16.84 dB, and 18.19 dB, respectively. The difference between the MG of B7M4 and the sum of the MG values of B7 and M4 was only 0.13 dB. Significantly, the 8-bit B5M4 and 10-bit B7M4 outperformed the 11-bit Barker code, demonstrating that BMOGCC achieves higher signal amplitude even with shorter code lengths. This is particularly advantageous in scenarios where shorter excitation signals are needed, for example, when DAQ memory is limited or boundary reflections interfere with useful signals [34]. These results further validate the superior performance of BMOGCC in enhancing the mainlobe amplitude.

**Figure 16:**
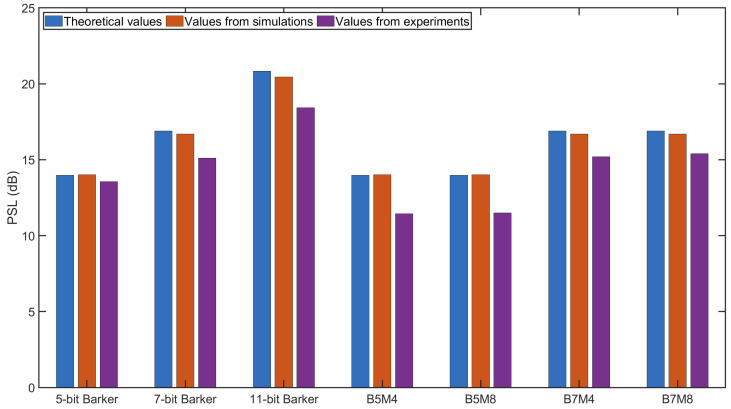
PSL values of different codes.

The PSL values obtained from both the simulations and experiments are shown in Figure 16, along with the theoretical values from Table 2. The PSL values from the simulations closely matched the theoretical expectations, with a maximum deviation of only 0.37 dB observed for the 11-bit Barker code. In the simulations, the PSL values of B5M4 and B5M8 were nearly identical to those of the 5-bit Barker code, while the PSL values of B7M4 and B7M8 were similar to those of the 7-bit Barker code. In the experimental results, the PSL values were lower than those of the simulations, with the largest discrepancy being 2.40 dB for the 11-bit Barker code. This variation was likely caused by the impulse response of the transducers and the quantization noise resulting from the low amplitude resolution of the DAQ system, both of which influenced the shape of the excitation signals and affected the PSL. Despite this, the PSL values observed in the experiments and simulations were generally consistent, confirming that BMOGCC provides sidelobe suppression comparable to that of the Barker code used in its formation.

The temporal resolution, estimated by the full width at half maximum (FWHM) of the decoded signal envelope, is shown in Table 6 for both the simulations and experiments. All decoded signals from simulations had a constant FWHM of 0.35 μs, regardless of the code type. The experimental FWHM values were higher and varied between 0.67 μs and 0.89 μs, mainly due to the transducer impulse response. Importantly, the results showed that BMOGCC maintained comparable temporal resolutions to those of the 1-bit pulse and other codes, confirming that it does not degrade the temporal resolution.

Overall, the results from both the simulations and experiments confirm the effectiveness of BMOGCC as the excitation signal for ultrasonic measurements.

- BMOGCC retains the low sidelobe levels of Barker codes and the double time efficiency of MOGCC, offering a key advantage over CGC and BCGC, which require two sequential transmissions.
- BMOGCC also provides a higher mainlobe amplitude than both Barker codes and MOGCC, even at shorter lengths, allowing more flexibility in signal design.
- Finally, BMOGCC preserves the temporal resolution, making it suitable for imaging applications where resolution is critical.

## 6. Conclusions

This study presented a novel coded excitation method, the Barker-convolved mutually orthogonal Golay complementary code (BMOGCC), designed to improve both time efficiency and signal quality in ultrasonic NDT. The proposed method is based on convolving the Barker code with MOGCC, combining the beneficial properties of both. BMOGCC retains the low sidelobe level of the Barker code and the simultaneous transmission capability of MOGCC, thereby doubling the time efficiency compared to traditional complementary Golay codes. In contrast to the Barker code, BMOGCC provides a greater signal amplitude and allows for flexible code-length selection, enhancing adaptability in practical measurements. The method was validated through both numerical simulations and laboratory experiments involving BMOGCC, the Barker code, MOGCC, and the 1-bit pulse. The results showed strong agreement between the simulations and experimental data. The findings demonstrated that the PSL of BMOGCC was comparable to that of the Barker codes used in its construction, confirming effective sidelobe suppression; BMOGCC achieved a significantly higher mainlobe amplitude than the other tested codes, even when using shorter sequences, indicating enhanced signal energy; and BMOGCC maintained the temporal resolution without any degradation compared to other coded excitation signals, ensuring its suitability for high-resolution ultrasonic imaging applications. Overall, BMOGCC shows excellent performance and provides a practical advancement in the field of coded excitation for ultrasonic testing, particularly in applications requiring high energy or time-efficient measurements.

One limitation of the proposed coded excitation method is that only two excitation signals can be transmitted simultaneously due to the inherent structure of BMOGCC codes. This constraint limits its broader applicability in array transducer systems with a larger number of elements. Future work should focus on developing convolved codes that enable the simultaneous transmission of multiple excitation signals. Moreover, the present study concentrated on ultrasonic compression waves, which are non-dispersive. Future research should be extended to investigate dispersive wave modes. Additionally, the influence of transducer characteristics on the performance of coded excitation signals warrants further detailed investigation.

## Figures and Tables

**Table 1:** Barker codes.

Coded Sequence Notation	$N_{B}$	Code Bit Value
B2	2	1,1 or 1,−1
B3	3	1,1,−1
B4	4	1,1,1,−1 or 1,1,−1,1
B5	5	1,1,1,−1,1
B7	7	1,1,1,−1,−1,1,−1
B11	11	1,1,1,−1,−1,−1,1,−1,−1,1,−1
B13	13	1,1,1,1,1,−1,−1,1,1,−1,1,−1,1

**Table 2:** MOGCC.

Coded Sequence Notation	Code Bit Value
$M4X_{1}$	1,−1,−1,−1
$M4Y_{1}$	−1,1,−1,−1
$M4X_{2}$	−1,−1,1,−1
$M4Y_{2}$	1,1,1,−1
$M8X_{1}$	1,1,1,−1,1,1,−1,1
$M8Y_{1}$	1,−1,1,1,1,−1,−1,−1
$M8X_{2}$	1,1,1,−1,−1,−1,1,−1
$M8Y_{2}$	1,−1,1,1,−1,1,1,1

**Table 3:** PSL and MG values of codes.

Code	Length	PSL	MG
Barker	$N_{B}$	$20\log_{10}\left(N_{B}\right)$	$20\log_{10}\left(N_{B}\right)$
CGC	$N_{C}$	-	$20\log_{10}\left(2N_{C}\right)$
MOGCC	$N_{M}$	-	$20\log_{10}\left(2N_{M}\right)$
BMOGCC	$N_{B}+N_{M}-1$	$20\log_{10}N_{B}$	$20\log_{10}\left(2N_{B}N_{M}\right)$

**Table 4:** Steel properties.

Young’s Modulus	Poisson Ratio	Density
195 GPa	0.3	$7910\;{\mathrm{kg}/m}^{3}$

**Table 5:** Excitation signals for sources.

Simulation No.	Code	Source 1	Source 2
1	-	1-bit pulse	-
2	Barker	B5	-
3	Barker	B7	-
4	Barker	B11	-
5	M4	$MX_{1}4$	$MY_{1}4$
6	M4	$MX_{2}4$	$MY_{2}4$
7	M8	$MX_{1}8$	$MY_{1}8$
8	M8	$MX_{2}8$	$MY_{2}8$
9	B5M4	$B5MX_{1}4$	$B5MY_{1}4$
10	B5M4	$B5MX_{2}4$	$B5MY_{2}4$
11	B5M8	$B5MX_{1}8$	$B5MY_{1}8$
12	B5M8	$B5MX_{2}8$	$B5MY_{2}8$
13	B7M4	$B7MX_{1}4$	$B7MY_{1}4$
14	B7M4	$B7MX_{2}4$	$B7MY_{2}4$
15	B7M8	$B7MX_{1}8$	$B7MY_{1}8$
16	B7M8	$B7MX_{2}8$	$B7MY_{2}8$

**Table 6:** Temporal resolution for different excitation signals.

Excitation Signal	FWHM of Simulations (μs)	FWHM of Experiments (μs)
1-bit Pulse	0.35	0.75
5-bit Barker	0.35	0.89
7-bit Barker	0.35	0.78
11-bit Barker	0.35	0.78
M4	0.35	0.70
M8	0.35	0.69
B5M4	0.35	0.68
B5M8	0.35	0.67
B7M4	0.35	0.67
B7M8	0.35	0.68

## Data Availability

The data presented in this article are available on request from the authors.
